# 16S rRNA gene amplicon data from four composite gut-region samples of *Microcerotermes diversus* workers

**DOI:** 10.1016/j.dib.2026.113302

**Published:** 2026-10-01

**Authors:** Seyed Qasem Mousavi, Azam Amiri, Ali R. Bandani

**Affiliations:** a Department of Plant Protection, College of Agriculture and Natural Resources, University of Tehran, Karaj, Iran; b Environment and Biotechnology Research Division, Research Institute of Petroleum Industry, Tehran, Iran; c Department of Landscape Engineering, Faculty of Geography and Environmental Planning, University of Sistan and Baluchestan, Zahedan, Iran

**Keywords:** Termitidae, V3-V4 marker sequencing, Intestinal microbiota, Bacterial community profiling, Operational taxonomic units, Microbial ecology, Hindgut compartmentalization

## Abstract

This data article describes bacterial V3-V4 16S rRNA gene amplicon data from four anatomically defined composite gut preparations of *Microcerotermes diversus* workers. Termites were collected from infested *Morus nigra* branches in Shush, Khuzestan Province, southwestern Iran. Guts from 400 workers were dissected into four anatomical categories, and corresponding tissues were pooled to generate one composite DNA sample and one sequencing library per category. The amplicons were sequenced on an Illumina MiSeq platform using a 2 × 300-bp paired-end configuration. Public resources comprise quality-filtered paired-end FASTQ files in the NCBI Sequence Read Archive under BioProject PRJNA1328141, 82 representative partial 16S rRNA gene sequences in GenBank under accessions PX349529-PX349610, and processed OTU, taxonomic, diversity, and rarefaction records in Figshare, together with four pH records reported in this article. Sample identifiers T1-T4 link the anatomical definitions, sequence runs, processed outputs, tables, and figures. The archived reads and processed files can be used for sample-level reanalysis, taxonomic reclassification, workflow benchmarking, and harmonized comparison with independently generated termite microbiome data.

Specifications Table

**Table:** 

Subject	Microbiology: Microbiome
Specific subject area	Termite gut microbiome profiling using V3-V4 16S rRNA gene amplicon sequencing
Type of data	Quality-filtered paired-end reads (FASTQ.gz); representative partial 16S rRNA gene sequences (GenBank); processed OTU, taxonomic-abundance, alpha-diversity, beta-diversity, and ordination data (RAR archive); rarefaction records (CSV); pH records (table); figures (PNG). Filtered; analyzed; processed.
Data collection	Worker termites were collected from infested *Morus nigra* branches in Shush, Iran. Guts from 400 workers were dissected into four anatomical categories, and corresponding tissues were pooled to yield one composite DNA sample per category. The V3-V4 16S rRNA gene region was amplified with primers 341F/806R and sequenced on an Illumina MiSeq (2 × 300 bp). Provider-filtered read pairs were merged with FLASH v1.2.11 and clustered into 97%-identity OTUs using the UPARSE workflow in USEARCH v7.0.1090. Mothur v1.31.2 was used for alpha-diversity analysis, and QIIME was used to generate the beta-diversity distance matrices. For each anatomical category, pooled gut sections were homogenized in 500 µL of sterile 0.9% NaCl physiological saline.
Data source location	Biological material was collected on 1 November 2017 from black mulberry branches in Shush, Khuzestan Province, Iran (31.840111° N, 48.384250° E). Sequence data are hosted by NCBI, and processed data are hosted by Figshare.
Data accessibility	**Repository name:** NCBI Sequence Read Archive (SRA) **Data identification number:** BioProject PRJNA1328141; T1: SAMN51305519/SRR35386565; T2: SAMN51305520/SRR35386564; T3: SAMN51305521/SRR35386563; T4: SAMN51305522/SRR35386562 **Direct URL to data:**https://www.ncbi.nlm.nih.gov/bioproject/PRJNA1328141 **Instructions for accessing these data:** Public access without registration; paired FASTQ files are linked from each SRA run. **Repository name:** NCBI GenBank **Data identification number:** PX349529-PX349610 **Direct URL to data:**https://www.ncbi.nlm.nih.gov/nuccore/?term=PX349529:PX349610 **Repository name:** Figshare **Data identification numbers:**10.6084/m9.figshare.30108655; 10.6084/m9.figshare.30102493 **Direct URLs to data:**https://doi.org/10.6084/m9.figshare.30108655; https://doi.org/10.6084/m9.figshare.30102493 **Instructions for accessing these data:** Both records are public and downloadable without registration.
Related research article	None

## 1 Value of the Data

• The dataset provides publicly accessible V3-V4 16S rRNA gene amplicon records from four anatomically defined gut-region pools of *Microcerotermes diversus*. Each library was generated by pooling the corresponding tissue from 400 workers.
• Consistent T1-T4 identifiers link each anatomical category to its SRA run, representative sequences, pH record, processed tables, and figures.
• Researchers working on termite-associated microbiota, insect-microbe interactions, microbial ecology, and amplicon bioinformatics can use the archived reads and processed outputs as a sample-level reference resource.
• The quality-filtered paired-end reads can be reprocessed with alternative denoising, sequence-variant inference, OTU-clustering, taxonomic classification, and reference-database workflows; the deposited representative sequences can also be reclassified.
• The processed abundance tables, diversity outputs, and distance matrices can support workflow benchmarking and harmonized cross-dataset comparisons when differences in primers, amplified regions, sampling units, sequencing platforms, and analysis pipelines are considered.

## 2 Background

Termites maintain specialized gut microbial communities involved in lignocellulose conversion and nutrient cycling [1,2]. In higher termites (Termitidae), prokaryotic symbionts have prominent digestive roles because the gut flagellates common in lower termites are generally absent [1,2]. The alimentary canal is divided into anatomical regions that differ in pH, oxygen availability, redox conditions, and substrate characteristics, providing the ecological context for region-resolved microbiome datasets [[3], [4], [5]].

*Microcerotermes diversus* is a wood-feeding higher termite and an economically important structural pest in southwestern Iran. Previous work has addressed its foraging biology, management, and culturable cellulolytic gut bacteria [[6], [7], [8]]. Public 16S rRNA gene amplicon datasets are available for whole-worker *Microcerotermes crassus* and pooled guts from other subterranean termites [9,10], but they use different hosts and sampling units. The present dataset was compiled to preserve the mapping among four defined gut-region pools, their sequence libraries, processed microbiome outputs, and pH records, and to provide a citable resource for reanalysis and cross-dataset comparison.

## 3 Data Description

### 3.1 Repository organization and file inventory

The released dataset is distributed across NCBI SRA, NCBI GenBank, and two Figshare records. The paired-end SRA files are SRR35386565_1.fastq.gz and SRR35386565_2.fastq.gz for T1; SRR35386564_1.fastq.gz and SRR35386564_2.fastq.gz for T2; SRR35386563_1.fastq.gz and SRR35386563_2.fastq.gz for T3; and SRR35386562_1.fastq.gz and SRR35386562_2.fastq.gz for T4. Each file pair contains the forward and reverse reads from one composite library.

GenBank accessions PX349529-PX349610 comprise 82 representative partial 16S rRNA gene sequences. The first Figshare record [11] provides one file, Processed_OTU_Tables_and_Diversity.rar, which groups the processed OTU abundance, taxonomic, alpha-diversity, beta-diversity, and ordination outputs. The second Figshare record [12] provides one file, Rarefaction data.csv, containing rarefaction records for T1-T4. At the repository level, each Figshare record contains one downloadable file.

### 3.2 Gut-region definitions, pH records, and archived sequencing statistics

Sample T1 extended from the foregut to the posterior end of the midgut; T2 comprised hindgut sections P1-P2; T3 corresponded to hindgut section P3; and T4 comprised hindgut sections P4-P5 (Fig. 1). The retained pH records were 6.5, 7.5, 9.1, and 7.8 for T1-T4, respectively (Table 1). Table 1 also maps each anatomical category to its BioSample, SRA run, archived read-pair count, and base total.

**Fig. 1:**
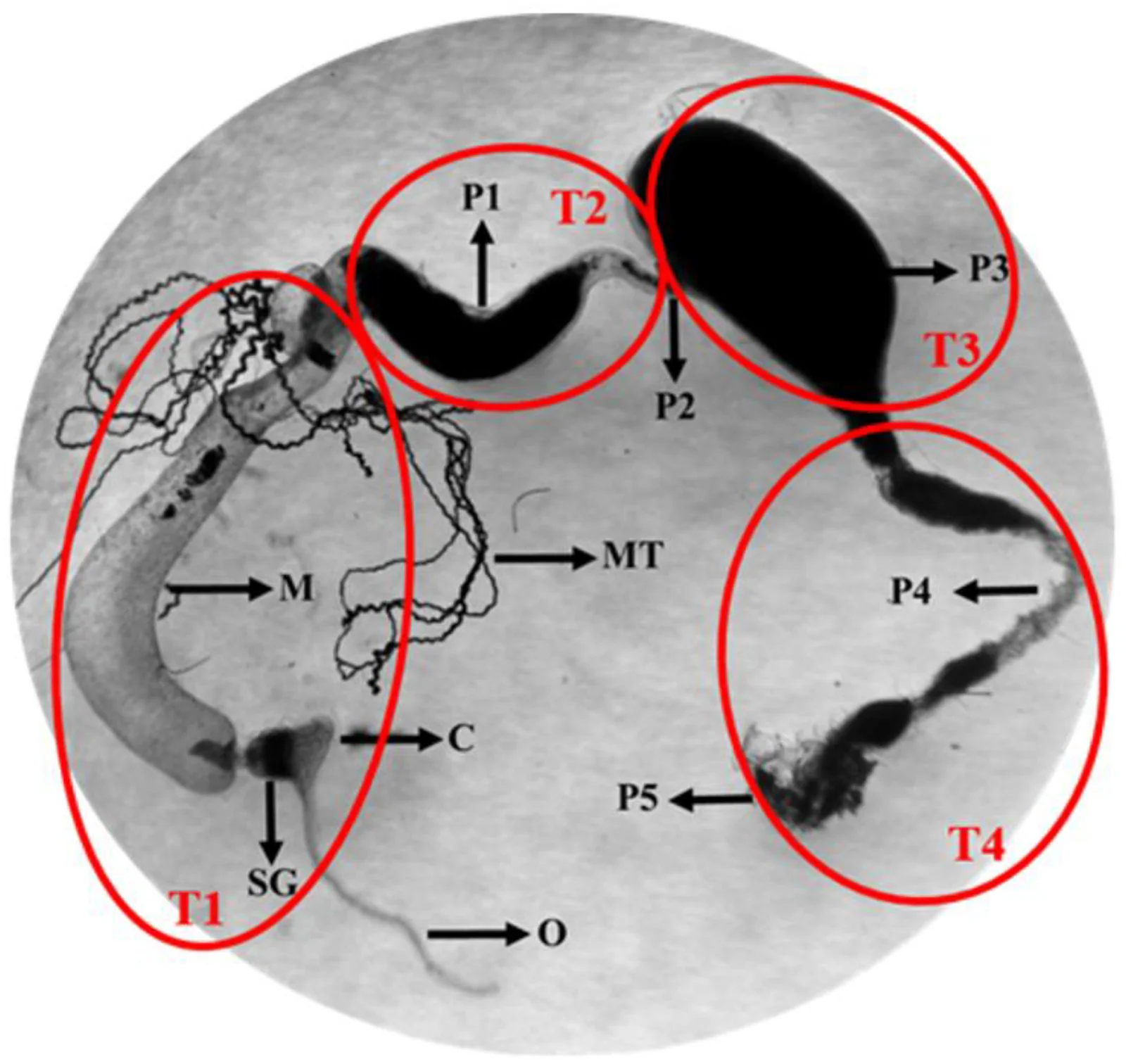
Anatomical definitions for the four composite gut categories. Gut morphology used to define T1-T4. O, esophagus; C, crop; SG, salivary glands; M, midgut; MT, Malpighian tubules; P1-P5, hindgut sections.

**Table 1:** Sample definitions, pH records, and publicly archived quality-filtered paired-end sequencing data for the four composite gut-region libraries of *Microcerotermes diversus*.

Sample	Anatomical pool	pH	BioSample	SRA run	Archived read pairs	Archived bases (Mbp)
T1	Foregut-posterior midgut	6.5	SAMN51305519	SRR35386565	63,077	36.180
T2	Hindgut P1-P2	7.5	SAMN51305520	SRR35386564	127,368	73.310
T3	Hindgut P3	9.1	SAMN51305521	SRR35386563	131,152	74.196
T4	Hindgut P4-P5	7.8	SAMN51305522	SRR35386562	88,494	50.705

Note: The pH column contains one retained value for each composite anatomical category. An archived read pair consists of one forward and one reverse read; read-pair and base totals correspond to the public ENA/SRA run records.

### 3.3 OTU and taxonomic-abundance data

Following paired-end merging and 97%-identity clustering, the processed abundance table contained 1978 operational taxonomic units (OTUs). Sample identifiers T1-T4 are retained across the SRA runs, processed tables, representative sequences, and visualizations.

Fig. 2 presents the relative-abundance profiles/stacked bar charts at phylum, family, and genus levels. The phylum-level output contained 19 categories; taxa below 0.5% were grouped as Other for visualization (Fig. 2a). The family-level and genus-level panels retain the corresponding unclassified categories (Fig. 2b,c). At the species-rank annotation level, >95% of sequences were unclassified. Any species-rank labels retained in the deposited processed outputs reflect the original pipeline annotations and should not be interpreted as validated species identifications because short V3–V4 amplicons do not provide sufficiently reliable species-level resolution across taxa. At a broad taxonomic level, the profiles are consistent with the strong gut compartmentation reported for wood-feeding higher termites. Firmicutes were prominent in T1 and T2, whereas T3 (P3) showed marked enrichment of Spirochaetes, particularly *Treponema*, together with Fibrobacteres, a pattern also reported for the P3 compartment of other wood-feeding higher termites. The increased representation of Bacteroidetes in T4 (P4–P5) is also consistent with the Bacteroidetes-rich P4 community reported in the wood-feeding *Microcerotermes parvus* [3,13]. These comparisons are descriptive because each anatomical category in the present dataset is represented by a single composite library.

**Fig. 2:**
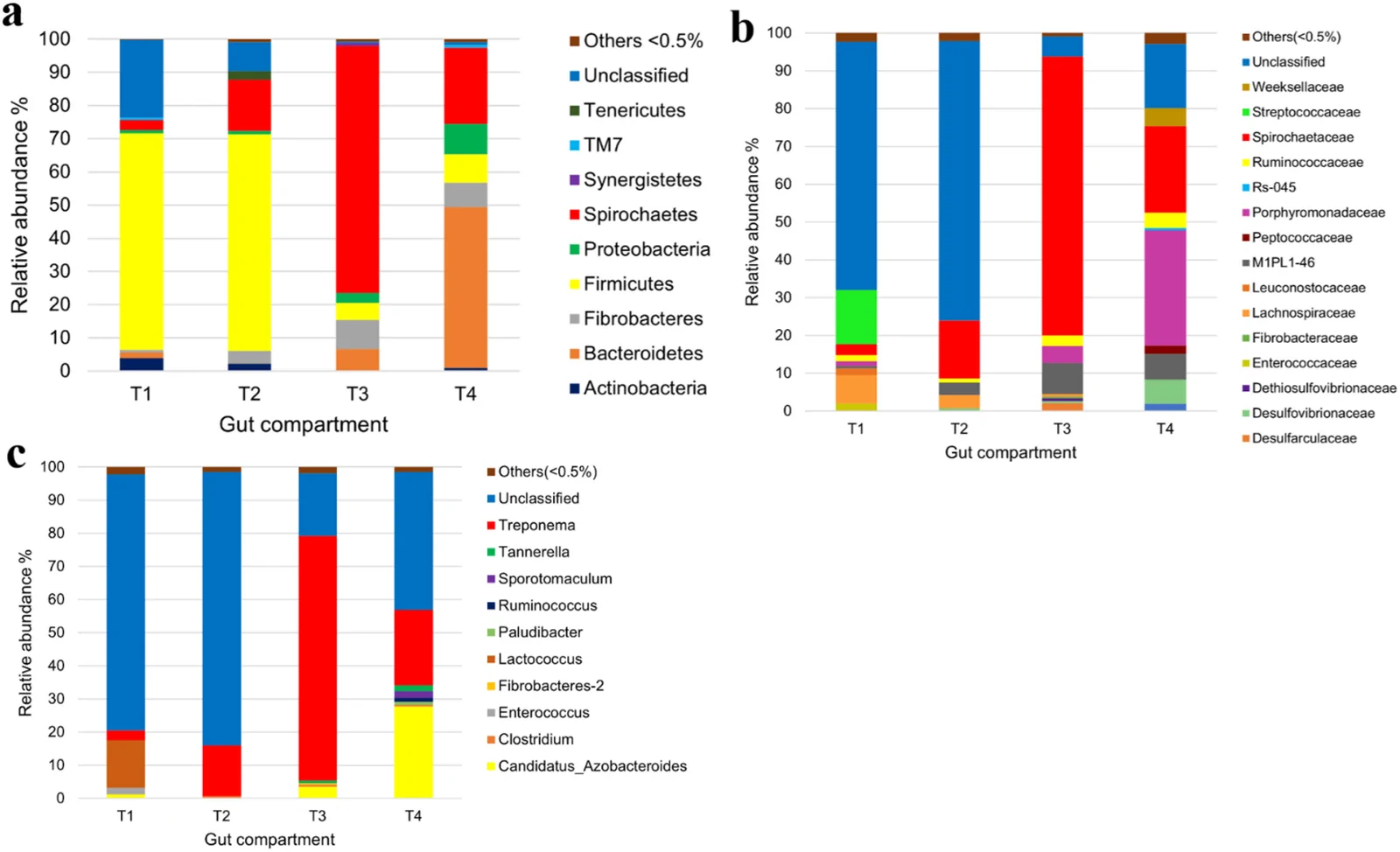
Relative abundance of bacterial taxa in the four composite libraries at (a) phylum, (b) family, and (c) genus levels. Categories with relative abundance below 0.5% were combined as Other. Values derive from one composite library per anatomical category.

### 3.4 Pairwise beta-diversity data

The processed outputs include pairwise Bray-Curtis, weighted UniFrac, and unweighted UniFrac distance matrices for the four composite libraries (Fig. 3a-c). Off-diagonal values range from 0.60 to 0.93 for Bray-Curtis dissimilarity, 0.51 to 0.98 for weighted UniFrac distance, and 0.25 to 0.47 for unweighted UniFrac distance. These matrices contain sample-level pairwise values and no replicate-based within-region estimates.

**Fig. 3:**
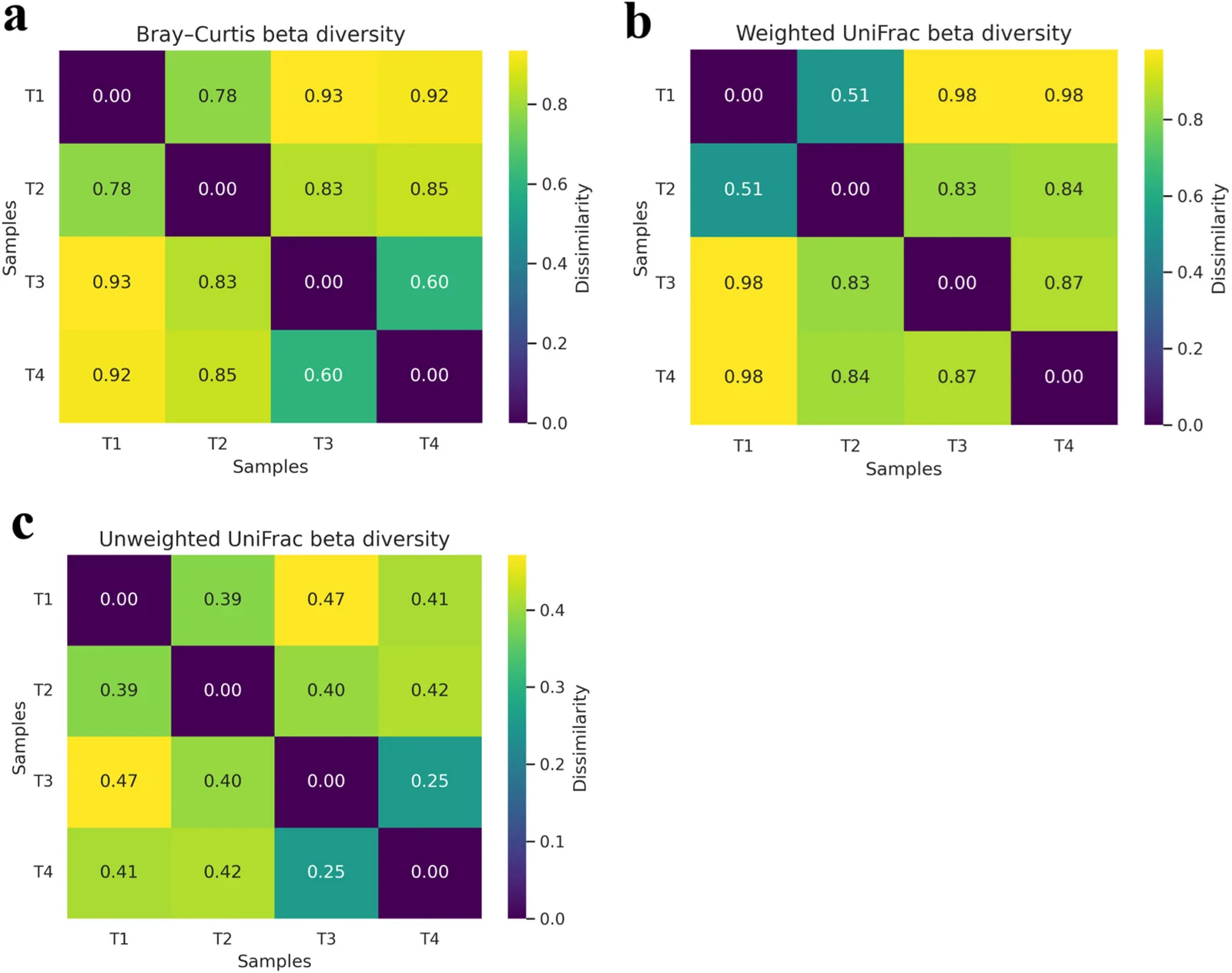
Pairwise beta-diversity distance matrices for the four composite libraries: (a) Bray-Curtis, (b) weighted UniFrac, and (c) unweighted UniFrac. Values are pairwise distances among T1-T4.

### 3.5 Alpha-diversity and rarefaction data

Table 2 provides one set of alpha-diversity estimates for each composite library. The values are single-library estimates rather than means or replicate-based summaries. Rarefaction data for observed OTUs are plotted against subsampled sequence count in Fig. 4.

**Table 2:** Alpha-diversity estimates for the four composite gut-region libraries of *Microcerotermes diversus*.

Sample	Sobs	Chao1	ACE	Shannon	Simpson
T1	464	514.7	528.8	3.67	0.05
T2	493	527.2	541.9	3.56	0.06
T3	516	551.5	562.7	3.61	0.07
T4	533	556.6	564.6	3.64	0.08

Note: Each value was calculated from one composite library. The values are not means, and no standard deviations or inferential statistical tests are reported.

**Fig. 4:**
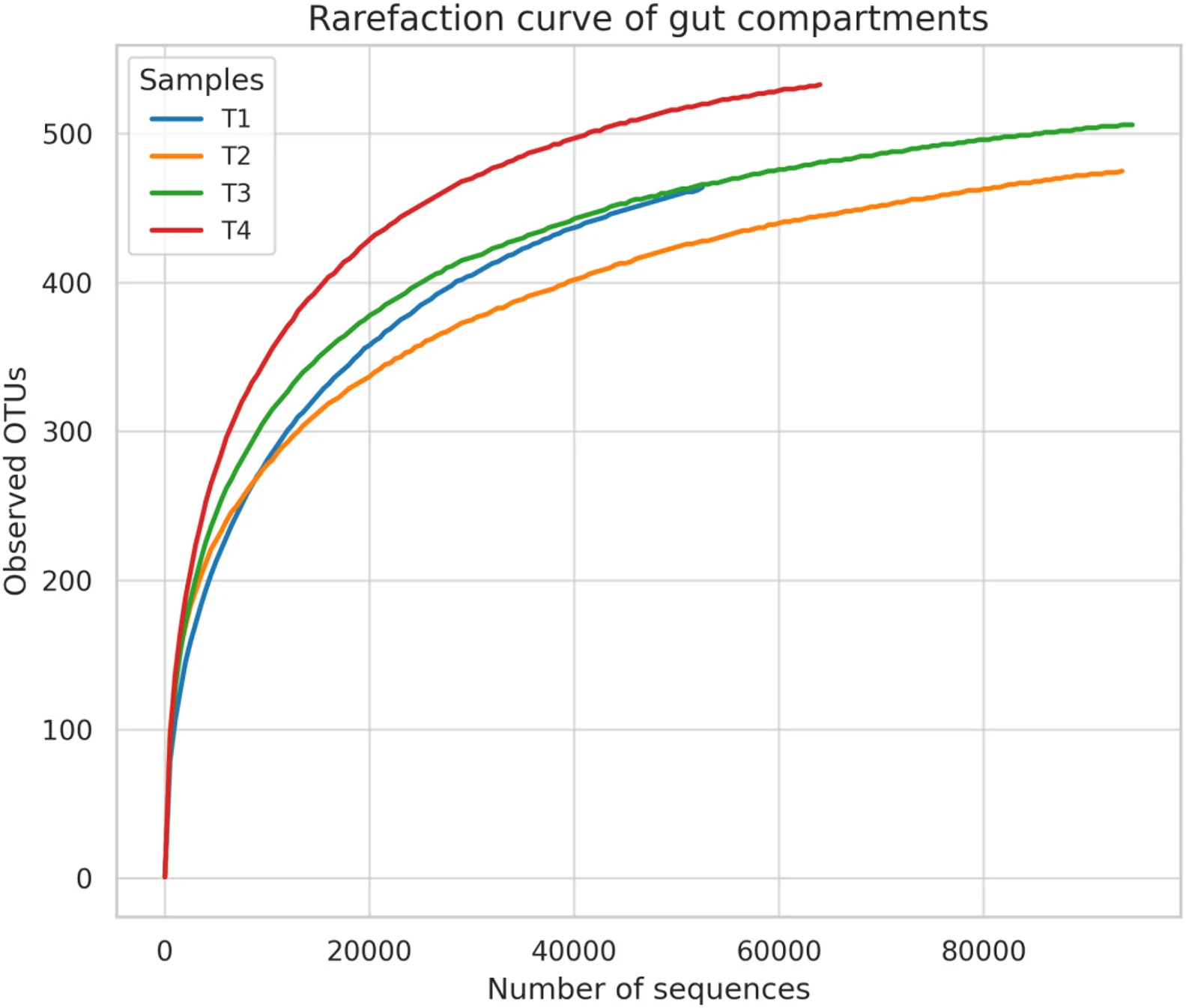
Rarefaction curves for observed OTUs in the four composite libraries. Curves plot observed OTUs against subsampled sequence count.

## 4 Experimental Design, Materials and Methods

### 4.1 Sample collection and composite-sample design

Worker termites were collected on 1 November 2017 from branches of black mulberry (*Morus nigra* L.) infested with *Microcerotermes diversus* in Shush, Khuzestan Province, southwestern Iran (31.840111° N, 48.384250° E). Infested branches approximately 30–50 cm long were removed from trees or collected from the ground and transported to the laboratory. The termite material was morphologically identified as *M. diversus* before worker selection and dissection.

Workers were obtained from multiple infested branches; branch- and colony-specific identities were not retained as independent sampling units. The branches were therefore not treated as biological replicates.

A total of 400 worker guts were processed. Each gut was divided into T1, extending from the foregut to the posterior end of the midgut; T2, comprising hindgut sections P1-P2; T3, corresponding to hindgut section P3; and T4, comprising hindgut sections P4-P5. Corresponding tissues from all 400 workers were pooled separately for each anatomical category, producing one composite DNA sample and one sequencing library for each of T1-T4.

### 4.2 Pre-dissection treatment, gut dissection, and pH recording

Before dissection, workers were held without food for 24 h. They were immobilized at −20 °C for 5 min and maintained on ice during preparation. External surfaces were cleaned with cotton moistened with 70% ethanol, followed by sterile distilled water. Guts were dissected under a stereomicroscope using sterile instruments and separated into the four anatomical categories.

For pH measurement, the corresponding gut sections from 400 workers were pooled separately for each anatomical category and homogenized in 500 µL of sterile 0.9% NaCl physiological saline. The pH of each pooled gut-region homogenate was measured immediately after homogenization using a labForce® pHE-08 semi-micro pH electrode (Thomas Scientific, USA). Before each measurement session, the electrode was calibrated at room temperature using standard pH 7.0 and pH 10.0 buffers. One retained pH value is available for each anatomical category.

### 4.3 Composite-sample preparation and DNA extraction

Each pooled gut-region preparation was homogenized with a sterile glass tissue grinder. Microbial DNA was extracted using the FastDNA Spin Kit (MP Biomedicals, USA) according to the manufacturer's instructions. DNA concentration and purity were assessed with a NanoDrop 2000 spectrophotometer (Thermo Fisher Scientific, Waltham, MA, USA), and DNA integrity was evaluated by electrophoresis on a 1% agarose gel. Extracted DNA was stored at −80 °C until amplification.

### 4.4 16S rRNA gene amplification and sequencing

The V3-V4 hypervariable region of the bacterial 16S rRNA gene was amplified using primer 341F (5′-CCTACGGGNGGCWGCAG-3′) and primer 806R (5′-GACTACHVGGGTATCTAATCC-3′) [14]. Amplicons were purified and quantified before sequencing, and one indexed library was prepared from each composite DNA sample.

Paired-end sequencing was performed by BGI, China, on an Illumina MiSeq platform using a 2 × 300-bp configuration. Sample identifiers T1-T4 were retained through sequencing and data deposition. No biological- or technical-replicate library, extraction-blank library, or PCR-negative-control library is included in the released sequence dataset.

### 4.5 Sequence processing and OTU construction

The sequencing-provider workflow removed adapter sequences and applied quality filtering with a Phred Q20 criterion. The SRA records contain the post-filtering paired-end files described above. Quality-filtered read pairs were merged with FLASH v1.2.11 [15], using a minimum overlap of 15 bp and a maximum mismatch ratio of 0.10 within the overlap.

Merged sequences were processed with the UPARSE workflow in USEARCH v7.0.1090 [16] and clustered into OTUs at 97% sequence identity. Read-to-OTU assignments were used to construct the deposited OTU abundance table. Dataset features are therefore reported as 97%-identity OTUs rather than amplicon sequence variants. The original OTU-based processing was retained to preserve consistency with the deposited processed dataset; the publicly archived quality-filtered reads remain available for independent reanalysis using contemporary ASV-based workflows.

Taxonomic labels produced by the original provider workflow were retained; no post hoc reclassification was performed for this data article.

### 4.6 Generation of descriptive microbiome data products

Alpha-diversity metrics were calculated from the 97%-identity OTU table at a distance label of 0.03 using mothur v1.31.2 [17]. The deposited outputs include observed OTUs (Sobs), Chao1 and ACE richness estimators, Shannon and Simpson indices, and rarefaction records.

Absolute taxonomic-abundance tables were generated by aggregating OTU counts at phylum, class, order, family, genus, and species-rank annotation levels. Species-rank labels were retained from the original provider pipeline and were not independently validated in this data article. Relative abundance for a category was calculated as its assigned count divided by the total assigned count for that sample and multiplied by 100.

Pairwise Bray-Curtis, weighted UniFrac, and unweighted UniFrac distance matrices were generated from the OTU-level data using the original QIIME workflow [18]. The alpha-diversity estimates and distance matrices were used only as descriptive outputs for the four composite libraries. No hypothesis test, PERMANOVA, differential-abundance analysis, pH-microbiome correlation, or functional prediction was performed.

## Limitations

The dataset contains four pooled V3-V4 16S rRNA gene amplicon libraries, with one composite library for each anatomical category. Corresponding tissues from 400 workers were combined for each category, and no independent biological- or technical-replicate libraries were retained. Pooling prevents assessment of individual-level variation, and the absence of replicate libraries prevents estimation of within-region variability or inferential comparison among regions. Accordingly, differences observed among T1–T4 should be interpreted as descriptive differences among the four composite libraries and not as estimates of biological differences among gut regions.

The pH data similarly contain one retained value per category. The public SRA files correspond to the quality-filtered read counts reported in Table 1; original pre-filtering FASTQ files are not included, so the earliest adapter-removal and quality-filtering steps cannot be repeated from the released files. Short V3–V4 amplicons do not provide sufficiently reliable resolution for confident species- or strain-level identification across taxa. Accordingly, species-rank labels in the deposited processed outputs should be regarded only as original pipeline annotations and not as validated taxonomic identifications. Marker-gene data do not directly measure gene content, expression, metabolic activity, or physiological function. No extraction blank or PCR-negative control library, shotgun metagenomic, metatranscriptomic, metabolomic, enzyme activity, cultivation, or targeted eukaryotic marker-gene data are included.

## Credit Author Statement

**Seyed Qasem Mousavi:** Investigation, Writing – original draft. **Azam Amiri:** Writing – review & editing. **Ali R. Bandani:** Conceptualization, Supervision, Project administration.

## Ethics Statement

The authors confirm that they have read and followed the ethical requirements for publication in Data in Brief. The work used worker termites (invertebrates) and did not involve human participants, vertebrate animal experiments, cephalopods, or data collected from social media platforms.

## Data Availability

(NCBI Sequence Read Archive (SRA)).Compartment-specific gut microbiome of Microcerotermes diversus (Original data)
